# Role of Melatonin in Bovine Reproductive Biotechnology

**DOI:** 10.3390/molecules28134940

**Published:** 2023-06-23

**Authors:** Zhiqiang Li, Kaiyan Zhang, Yuming Zhou, Jing Zhao, Jun Wang, Wenfa Lu

**Affiliations:** 1 Joint Laboratory of the Modern Agricultural Technology International Cooperation, Ministry of Education, Jilin Agricultural University, Changchun 130118, China; lizhiqiangsky@126.com (Z.L.); 17843096083@163.com (K.Z.); zhouyuming918@163.com (Y.Z.); jlndfztd@163.com (J.Z.); 2 Key Lab of Animal Production, Product Quality, and Security, Ministry of Education, Jilin Agricultural University, Changchun 130118, China; 3 College of Animal Science and Technology, Jilin Agricultural University, Changchun 130118, China

**Keywords:** melatonin, cattle, antioxidant, reproductive physiology

## Abstract

Melatonin has profound antioxidant activity and numerous functions in humans as well as in livestock and poultry. Additionally, melatonin plays an important role in regulating the biological rhythms of animals. Combining melatonin with scientific breeding management has considerable potential for optimizing animal physiological functions, but this idea still faces significant challenges. In this review, we summarized the beneficial effects of melatonin supplementation on physiology and reproductive processes in cattle, including granulosa cells, oocytes, circadian rhythm, stress, inflammation, testicular function, spermatogenesis, and semen cryopreservation. There is much emerging evidence that melatonin can profoundly affect cattle. In the future, we hope that melatonin can not only be applied to cattle, but can also be used to safely and effectively improve the efficiency of animal husbandry.

## 1. Introduction

Melatonin was discovered by Aaron Lerner in 1958 in the bovine pineal gland, and was determined to have an important role in the regulation of the circadian rhythm [1]. Consequently, melatonin became the “gold standard” drug for modulating the circadian rhythm [2] and was used to regulate sleep quality and body rhythms. Over the past 60 years, melatonin has been implicated in various tissues and cells in several animals [3,4,5,6], including the testes [7,8], ovaries [9], placenta [10], granulosa cells [11], and oocytes [12]. Melatonin has many roles in these tissues and cells, including scavenging reactive oxygen species (ROS) [13,14,15] and antioxidant [16,17], anti-apoptosis [18,19,20], anti-inflammatory [20,21,22], and anti-aging [23,24,25] activities.

The economic viability of the cattle industry depends heavily on good reproductive performance, which is affected by genetics, age, parity, body weight, nutrition [26], stress [27], endometritis [28], embryo quality [29], and conception rate [30]. Furthermore, bull breed and semen quality are also important factors affecting reproductive performance [31]. These factors can have causal relationships or may directly affect reproductive performance.

**Figure 1:**
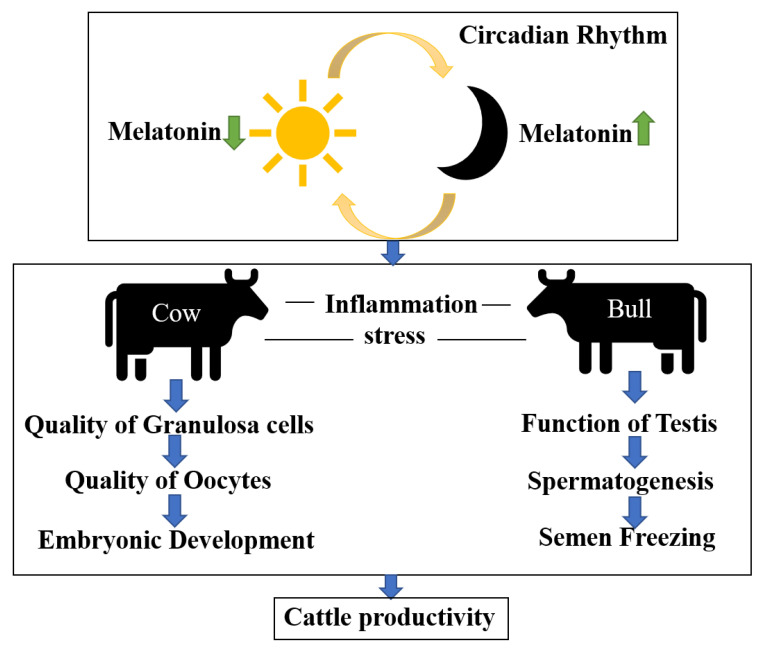
Effects of melatonin on physiological status of cattle.

Although studies of melatonin in cattle are rapidly accumulating, there has not been a systematic review of its effects on cattle reproductive performance. Therefore, the main objective of this review was to focus on various factors that can influence bovine reproduction and explore how melatonin can mediate these factors. This includes examining the effects of melatonin on granulosa cells, oocytes, circadian rhythms, stress, inflammation, testicular function, spermatogenesis, and semen cryopreservation (Figure 1). We aim to provide insights to increase the use of exogenous melatonin in livestock reproduction.

## 2. Application of Melatonin in Bovine Granulosa Cells

Follicle atresia is important in the decline in bovine reproductive performance, and the development of ovarian follicles relies heavily on granulosa cells. Any alteration in the state of these cells, including apoptosis, autophagy, cell cycle arrest, or an accumulation of ROS, can trigger follicular atresia [32,33,34]. In addition, changes in steroid hormone synthesis also affect granulosa cell states [35].

Melatonin is widely recognized for its ability to scavenge ROS and regulate cellular physiology; in granulosa cells, it can reduce ROS and inhibit apoptosis through various mechanisms [33,35]. During the early stage of follicular atresia, the inner granulosa cell layer undergoes apoptosis, whereas the cumulus–oocyte and outer granulosa cells do not undergo apoptosis [36]. Therefore, granulosa cell variation may be an important first step in follicular atresia. Granulosa cells have critical roles in maintaining and supporting the growth of follicles in vivo, with various physiological states determining follicle fate [37]. Alterations in key cellular functions, including those of mitochondria, have important consequences, including ROS release, which triggers apoptosis and autophagy. Mitochondria are one of the main sources of ROS. Whereas some O_2_ is used to produce ATP to maintain mitochondrial function, some will generate ROS, and in excess concentrations, this will cause extracellular Ca^2+^ to enter cells, causing mitochondrial swelling and even rupture. Melatonin can sustain antioxidant enzymes and eliminate active oxygen by regulating ER oxidoreductin1 (ERO1) to improve the activity of superoxide dismutase (SOD) and catalase (CAT) [18,38]. Furthermore, melatonin can counteract β-zearalenol-mediated oxidative stress and apoptosis in bovine ovarian granulosa cells, along with significant increases in SOD and CAT proteins [33]. Inhibition of the melatonin receptors MT1 and MT2 can abate the effects of melatonin and block changes in the cell cycle [32]. In addition to scavenging ROS, melatonin has different effects depending on temperature and O_2_ concentration. At 37.5 °C and 5% O_2_, which approximates in vivo conditions, a low melatonin concentration promoted cell proliferation, but at 40 °C, a high melatonin concentration promoted cell proliferation [39]. Therefore, melatonin interacts with temperature in a dose-dependent manner, with the potential for melatonin to reduce stress caused by high temperatures. However, the body’s physiological status and melatonin secretion vary, posing challenges for safe and effective application of exogenous melatonin, emphasizing the need for reliable data to make evidence-based decisions.

## 3. Application of Melatonin in Bovine Oocyte Cells

Oocyte quality is a crucial factor affecting the reproductive performance of female animals and a key limiting factor in ruminant embryo transfer. Live birth rates were significantly higher for the transfer of fresh versus cryopreserved embryos [40]. Oocyte cryopreservation has been a longstanding challenge due to factors such as rates of survival, fertilization, and development [41]. Therefore, it is necessary to improve the quality of oocytes cultured in vitro prior to cryopreservation, with melatonin having considerable promise.

Numerous studies have demonstrated that melatonin can improve the developmental capacity of oocytes both in vitro and in vivo [42]. For instance, it enhances oocyte developmental competence and embryonic development in prepubertal and adult cattle by mitigating ROS [43]. Melatonin reduces the ROS content of heat-shocked oocytes, increases oocyte maturation rate and the proportion of embryos that develop into blastocysts, and increases the transcriptional abundance of genes related to mitochondrial function [44]. Additionally, melatonin also protects bovine oocytes from other harmful substances, e.g., preventing paraquat-induced oocyte damage and preserving embryonic developmental capacity [45]. There is abundant evidence for the ability of melatonin to promote bovine oocyte development [46]. In this regard, melatonin promotes the synthesis of antioxidant enzymes via specific membrane or nuclear receptors to remove ROS. Acetylserotonin O-methyltransferase (ASMT) in cumulus–oocyte complexes (COCs) may be involved in melatonin synthesis [47]. Melatonin reduces the oxidative stress of oocytes through the MT1 membrane receptor, protecting the spindle body function to maintain oocyte development. In Holstein cows, 20 mg of melatonin on days 190–262 of gestation increased uterine blood flow, possibly due to its effect on steroid metabolism [48]. Melatonin also altered estradiol metabolism to improve uteroplacental development in heifers [49]. In our studies of estrus and artificial insemination in cattle, exogenous melatonin significantly increased progesterone concentrations, enhanced SOD, CAT, and glutathione peroxidase (GSH-Px) activity, and decreased MDA concentrations in cattle blood, with a significant increase in pregnancy rate [50]. Melatonin could be a valuable tool for improving oocyte and embryonic development in vitro and a means to enhance in vivo fertility.

## 4. Melatonin Regulates Circadian Rhythms in Cattle

Melatonin can regulate behavior and reproduction by controlling the expression of genes involved in the circadian rhythm [51]. In addition, circadian rhythms can also affect cow physiology [52]. For example, in one study, luteinizing hormone (LH) secretion peaks before ovulation appeared 2–3 times more often at night than during daylight hours, whereas melatonin secretion peaked at night [53]. Interestingly, in luteal cells, melatonin increases the secretion of gonadotropin-releasing hormone (GnRH) and LH, thus enhancing progesterone secretion in a dose-dependent manner. Whereas granulosa cells are critical for estrogen secretion, luteal cells secrete more progesterone [54]. Perhaps melatonin directly affects ovulation in animals. For example, the influence of melatonin on GnRH and LH is blocked by luzindole, an inhibitor of the melatonin receptors MT1 and MT2. However, further investigation is needed to confirm the effects of melatonin on ovulation.

Melatonin also affects cow performance, as inhibiting melatonin secretion with light increases milk production in high-producing cows [55]. Long-day exposure reduces blood melatonin concentrations and increases blood prolactin concentrations in cattle [56,57], whereas exogenous melatonin suppresses prolactin [58]. Consequently, melatonin has profound effects on dairy cow performance, with the potential to modulate cow physiology and performance with light control and exogenous melatonin. Photoperiod has been used to improve animal productivity, including in chickens [59], sheep [60], and horses [61].

Melatonin is considered the “switch” that regulates circadian rhythms. One of the core genes of the circadian rhythm is BMAL1, which is considered indispensable. BMAL1 is in various cells and tissues, including the liver, testes, oocytes, and granulosa cells [62]. If damaged, it will cause the body to have a series of physiological abnormalities, including abnormal sleep, abnormal ovulation, and a shortened lifespan [63]. Melatonin restores sleep disorders in Parkinson’s disease (PD) patients by promoting BMAL1 gene expression. [64]. Furthermore, melatonin not only enhances autophagy through BMAL1 but also improves cerebral ischemia–reperfusion in diabetic mice [65]. It can also increase the expression of clock proteins to affect endocrine status and improve obesity in mice [66]. Blocking the BMAL1 gene with siRNA reduced the effects of melatonin on rooster circadian rhythms, implying that melatonin primarily functions through BMAL1 [67], with BMAL1 at the apex of the circadian clock feedback pathway in the avian retina [68]. Melatonin can regulate the secretion of testosterone and progesterone in male and female animals, which may be achieved through the core gene BMAL1. Despite no direct evidence to support this conjecture, reducing the expression of BMAL1 reduced testosterone secretion [69], and BMAL1 knockout mice had delayed genital development and puberty [70], perhaps due to the effects of melatonin on testosterone and progesterone secretion. Although most effects of circadian rhythms on cattle are centered on photoperiod, there is also evidence that BMAL1 has an important role in cattle. Knockdown of BMAL1 decreased prostaglandin F2α (PGF2α) in bovine uterine stromal cells (USCs) [71]. There is also evidence that BMAL1 functions as a core gene in bovine oocytes and preimplantation embryos to perform the same function as maternal mRNA [72]. In addition, circadian rhythm genes are involved in regulating neutrophil functions, helpful for assessing perinatal disease susceptibility in cattle [73]. Clearly, photoperiod and circadian rhythms affect cattle reproduction, with further studies needed to determine the specific roles of melatonin and mechanisms of action.

## 5. Effects of Melatonin on Inflammation in Cattle

Melatonin is a “regulator” of the immune system with both pro- and anti-inflammatory effects [74]. However, melatonin’s role in inflammation is not innate, but it depends on specific conditions. Melatonin generally has antioxidant and anti-inflammatory functions in most cells to maintain homeostasis [75,76]. In contrast, in tumor and cancer cells, it has potent pro-oxidative and pro-apoptotic therapeutic effects [77,78].

Exogenous melatonin can be given to treat various diseases by reducing inflammation and oxidative stress [74]. For example, it attenuated metabolic inflammation in mice by increasing exosomal α-ketoglutarate (αKG) [79]. It can also alleviate secondary brain injury caused by cerebral hemorrhage in rats by inhibiting inflammation [21]. Additionally, exogenous melatonin inhibited the release of inflammatory factors IL-6, IL-1β, and TNF-α in human nucleus pulposus cells (NPC), thereby suppressing inflammation [22]. Although less research has been conducted on the role of melatonin in bovine inflammation, it enhanced endometrial receptivity by alleviating ammonia-induced inflammation and apoptosis through the TLR4/NF-κB/IL-6 signaling pathway [80]. Rumen bypass membrane feeding (RBMF) can inhibit stress response and inflammation in dairy cows [81]. Melatonin also improved *Staphylococcus aureus*-induced mastitis by acting on the Microrna-16B/YAP1 pathway [82]. In addition, exogenous melatonin improved the efficiency of the bovine viral diarrhea virus vaccine [83], and based on in vitro studies, it inhibited NF-κB activity and reduced IL-1β and IL-6 mRNA levels [83]. Furthermore, melatonin also enhanced the immune response of sheep inoculated with *Dichelobacter nodosus* and increased serum IgG concentrations [84]. Therefore, melatonin has some anti-inflammatory and antiviral effects, with the potential to prevent or treat cattle diseases.

## 6. Effects of Melatonin on Testicular Function, Spermatogenesis, and Semen Cryopreservation in Bulls

Cryopreservation is an important assisted reproductive technology for long-term gamete preservation in ruminants such as cattle and sheep [85]. In addition to the importance of oocytes, high-quality semen also has an important role in accelerating genetic improvement, and is important for sustaining endangered animals. Therefore, there is much impetus to improve semen cryopreservation technology.

Semen contains a certain concentration of reactive oxygen species that are necessary for sperm capacitation [85]. However, high concentrations of ROS can cause oxidative stress to damage sperm physiological functions, including morphology and DNA integrity [86]. Melatonin reduces ROS-induced oxidative stress during sperm freezing and maximizes the quality of frozen-thawed sperm due to its ROS scavenging ability [10,87,88].

Adding 1 mM melatonin to semen extender improved the quality of swamp buffalo sperm [89]. Adding melatonin to semen extender mitigated reductions in the quality of frozen-thawed sperm from heat-stressed rams [90]. In Murrah buffalo bulls, a melatonin implant (18 mg/50 kg of body weight) lasted for 2 months and significantly reduced morphologically abnormal sperm and increased sperm motility, both in terms of curve and linear velocity. In addition, melatonin increased the concentration of total protein and cholesterol in seminal plasma and improved the semen quality of Murrah buffalo bulls during the non-breeding season under tropical conditions [91]. 

Su et al. [92] systematically studied effects of melatonin on semen cryopreservation, oocyte maturation, and embryonic development. During these processes, the optimal concentration of melatonin was not consistent. During semen freezing, 10^−3^ M was optimal, whereas 10^−7^ M significantly increased the oocyte maturation rate and also increased the total number of blastocysts in in vitro fertilization (IVF). In our previous work, various concentrations of melatonin had different effects on sperm motility and antioxidant indicators. Both low (0.125 mg/mL) and high (0.5 mg/mL) melatonin concentrations reduced ROS content. In addition, a medium concentration (0.25 mg/mL) of melatonin reduced MDA content. Although all three concentrations of melatonin improved antioxidant indicators, the medium concentration had the best advantage [87]. In conclusion, adding an appropriate concentration of melatonin to semen extender and sperm preparation for in vitro fertilization can improve the quality of frozen-thawed sperm, embryonic development, and success of in vitro fertilization.

In addition to exogenous melatonin in extender improving semen quality, melatonin is a key factor in spermatogenesis, which regulates testicular function through the hypothalamic–pituitary–gonadal axis [93,94]. The hypothalamic–pituitary–gonadal (HPG) axis is key to regulating reproductive hormones. Puberty was delayed in pups in pregnant female rats given melatonin, attributed to decreased LH and prolactin [95,96]. In addition, melatonin inhibits GnRH-induced LH release, thereby inhibiting testosterone production, whereas luzidole, an inhibitor of melatonin receptor MT1, essentially counteracts the effects of melatonin [96,97,98]. During testicular growth and development, melatonin has a critical role in several testicular cell types and hormone secretion. Melatonin protects the testes from local inflammation and reactive oxygen species [93,99,100]. Male reproductive function depends on the HPG axis, and melatonin can affect hormone synthesis, e.g., by modulating the growth and development of several testicular cells through its receptor [101]. Melatonin released by the pineal gland can also be absorbed by the testes through blood circulation, thereby modulating testicular activity [93]. In addition, melatonin can act through its unique receptors to regulate testosterone secretion, apoptosis, and autophagy [7,102,103]. Melatonin treatment of bovine Sertoli cells in vitro increased the expression of genes related to spermatogenesis, including Cyclin D1, Cyclin E, Pdgfa, Dhh, Occludin, and Claudin [104]. After healthy men took melatonin for 6-month, there were changes in some aspects of their semen, perhaps due to melatonin-induced inhibition of aromatase [105]. In a testicular ischemia–reperfusion model, melatonin significantly reduced morphologically abnormal sperm [106]. In vitro, melatonin increased the percentage of motile sperm and increased mitochondrial activity, implying that melatonin affected spermatogenesis and development through the blood–testis barrier [107]. These findings confirmed that melatonin regulated spermatogenesis via the development of testicular cells.

## 7. Positive Effects of Melatonin in Livestock Cells

Many positive effects of melatonin have been described above. However, optimal concentrations of melatonin seem to differ according to tissues or cells of various species. Due to relatively long estrous cycles (~21 days) and pregnancy (~285 days), it can be technically challenging to study the effects of melatonin in cattle. To the best of our knowledge, we were the first to report that melatonin improved fertility in cows [50]. Moreover, melatonin regulates the circadian rhythm and has a vital role in the milk production and metabolism of lactating cows [108]. The HPA axis can also promote gonadal atrophy of dairy cows in winter by increasing the secretion of melatonin [109]. There are limited studies on melatonin in domestic livestock, with a variety of doses and outcomes (Table 1). Melatonin can directly remove free radicals and ROS, and improve the antioxidant capacity by regulating cosmodal peroxidase and glutathione reduction [110]. 

Melatonin can also mitigate body damage nitrogen peroxide compound enzymes and nitrogen-based poison [111]. Melatonin can interact with heavy metals [112], combining with iron and copper. In hemoglobin, melatonin can restore iron (Feiv-O) to Iron (III) [113]. Melatonin can also be combined with CU (II) and CU (I) to reduce the lipid peroxidation of copper mediated in the liver [114]. Furthermore, melatonin can also assist mitochondria to clear free radicals and ROS [115]. Mitochondria are the site of ATP and ROS production [116]. The core theory of aging is mitochondrial damage, and melatonin can target mitochondria to generate ATP, remove ROS, and delay aging [117].

Melatonin can improve human sleep quality [118], regulate cardiovascular [119], central system [120], nervous system [121], and immune system function [122], and prevent the occurrence of some diseases to a certain extent, including Parkinson’s disease [123], Alzheimer’s disease [124], depression [125], and ischemic brain injury [126]. These human studies demonstrate the safety and effectiveness of melatonin, which lays the foundation for its use in animal husbandry, where there is much potential.

**Table 1:** Positive effects of melatonin on cattle, sheep, and pigs.

Species	Positive Effects of Melatonin	Concentration	References
Cattle	Melatonin promoted diameter of bovine follicles and growth of secondary oocytes	10^−7^ M	[127]
	Melatonin in cattle feed changed the β diversity of vaginal microorganisms	20 mg	[128]
	Melatonin promoted proliferation of bovine theca cells and inhibited steroid production	1 μM	[129]
	Melatonin promoted bovine oocyte development and maturation	10^−7^ M	[12]
	Melatonin inhibited oxidative stress and apoptosis of bovine granulosa cells	100 μM	[33]
	Melatonin increased conception rates in cattle	0.24 mg/kg	[50]
	Melatonin decreased ROS production in bovine sperm and increased sperm viability, plasma membrane integrity, mitochondrial integrity, and acrosome integrity	10^−3^ M	[92]
	Melatonin promoted development and function of bovine Sertoli cells	320 pg/mL	[104]
Pig	Melatonin regulated lipid metabolism in porcine oocytes	10^−9^ M	[130]
	Melatonin reduced ROS production in porcine oocytes and promoted mitochondrial function and embryonic development	500 nmol/L	[131]
	Melatonin improved the quality of porcine embryos	1 nM	[132]
	Melatonin improved semen viability and acrosome integrity in pigs	1 μM	[133]
	Melatonin regulated ATP metabolism and antioxidant enzyme activity of boar sperm	1 μM	[134]
Sheep	Melatonin inhibited LPS-induced inflammation of sheep epididymal epithelial cells	10^−7^ M	[135]
	Melatonin was involved in activation of primordial follicles in sheep ovaries	100 pg/mL	[136]
	Melatonin promoted development of transgenic sheep embryos and improved transgenic efficiency	10^−7^ M	[137]
	Melatonin reduced ROS accumulation in sheep testicular interstitial cells and promoted testosterone synthesis	10 ng/mL	[138]
	Dietary supplementation of melatonin increased activities of glucose amylase, isomaltase, and maltase in small intestine of sheep	5 mg/d	[139]
	Melatonin reduced ROS and improved sperm quality	10^−7^ M	[140]
	Melatonin enhanced DNA integrity and fertilization ability of sheep sperm	1 mM	[141]

## 8. Conclusions and Prospects

The physiological states of domestic animals will affect their reproductive function. Melatonin has important roles in the growth, development, and metabolism of various cells, but is rarely studied in livestock. This review emphasizes how melatonin works and its potential for use in animal, but much remains unknown.

**Figure 2:**
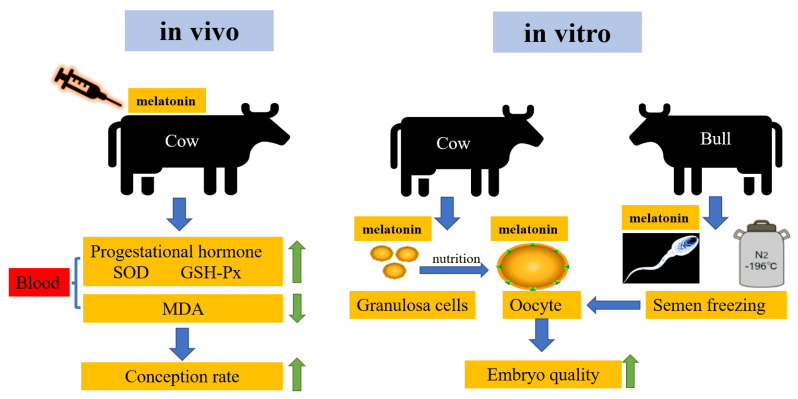
Effects of melatonin on bovine reproduction.

In female livestock, melatonin can promote ovulation, enhance ovarian cell development and embryonic development, enhance placenta development, and increase pregnancy rate. In male livestock, melatonin can enhance testicular function, improve sperm morphology and motility, improve protein content in sperm, and enhance mitochondrial activity (Figure 2).

Improving the efficiency of livestock reproduction is critical for improving the sustainability of cattle reproduction. However, there are various factors affecting the breeding of domestic livestock. The physiological state of livestock and its interaction with the environment are complicated. As expected, melatonin has become a human health product, and its safety has been recognized. However, it is limited to basic research on domestic animals, which is even more rare for improving reproduction efficiency and has certain limitations. Melatonin as an endogenous hormone is important in the field of animal science and progress should be made in large livestock animals. We expect that in the future, scientific researchers can use melatonin to change domestic animal physiology (e.g., endocrinology, metabolism, and estrus cycle) and pathology. The combination of melatonin and scientific breeding management may have the best effect, thereby improving the reproductive performance of domestic animals.

## Data Availability

Not applicable.
